# Variants loci and phenotype correlation of *TRIM8-*related neuro-renal syndrome: three cases reports and literature review

**DOI:** 10.3389/fneur.2024.1410187

**Published:** 2024-10-01

**Authors:** Qiu Lv, Yue Niu, Zhao Xu, Jiong Qin, Zhixian Yang

**Affiliations:** ^1^Department of Pediatrics, Peking University People’s Hospital, Beijing, China; ^2^Epilepsy Center, Peking University People’s Hospital, Beijing, China

**Keywords:** *TRIM8*, neuro-renal syndrome, epilepsy, developmental delay, nephrotic syndrome, proteinuria

## Abstract

**Background:**

*TRIM8*-related neuro-renal syndrome (NRS), caused by pathogenic variants of the *TRIM8* gene, is characterized by epilepsy, developmental delay (DD) and renal disorders. The severity of the neurological effects as well as the presence of renal disorders is variable among patients. Here, we report three additional patients with clinical features compatible with NRS and summarize the association between the variants’ loci and phenotype of *TIRM8*-related NRS.

**Methods:**

A retrospective analysis was conducted for three Chinese children with NRS due to *TRIM8* variants identified through whole-exome sequencing (WES). Previous reports of patients with *TRIM8*-related NRS were reviewed systematically. Demographic and clinical data were collected from these patients

**Results:**

Two *de novoTRIM8* truncating variants in three NRS patients were identified in our study, including c.1327_c.1328delCCinsTG (p. Arg443*) and c.1375C>T (p.Gln459*). Our three patients all exhibited drug-resistant epilepsy and early-onset DD, and two of whom developed electrical status epilepticus during sleep (ESES). Brain magnetic resonance imaging (MRI) showed periventricular leukomalacia in one patient and normal in the other two. All three patients demonstrated nephrotic range proteinuria (NRP) or nephrotic syndrome (NS) with normal renal function during follow-up. There was a total of 27 patients with *TRIM8*-related NRS have been identified to date. The most common clinical features are renal diseases (89%), DD (89%), followed by epilepsy (78%). 67% of patients eventually progressed to end-stage renal disease (ESRD). Focal seizure was the most frequent seizure type (57%). 52% of patients presented drug-resistant epilepsy. 64% of patients exhibited non-specific brain MRI abnormalities. Brain atrophy was the most common change (50%). Two patients with TRIM8 variants closer to the N-terminal had neurological diseases without renal damage. Five patients with *TRIM8* variants closer to the C-terminal had no severe neurological diseases. Seven patients had Gln459* variant which is the most common variant (7/27, 25.9%). The severity of the renal and neurological damage of the seven patients was variable.

**Conclusion:**

This study expands the number of individuals with confirmed NRS due to pathogenic variants in *TRIM8*. Neurological and renal phenotype with the same variant locus differed in their severity. Further research is needed to explore the relationship between genotype and phenotype of *TRIM8* variants.

## 1 Introduction

Truncating variants in *tripartite motif containing 8(TRIM8)* gene (OMIM:606125), firstly described by Allen et al. in 2013 (1), are the genetic basis of neuro-renal syndrome (NRS) encompassing renal and neurodevelopmental phenotype (2). The disorder shows an autosomal dominant pattern of inheritance. Renal phenotype includes asymptomatic proteinuria, nephrotic-range proteinuria (NRP), nephrotic syndrome (NS), focal segmental glomerulosclerosis (FSGS) and most cases eventually progress to end-stage renal disease (ESRD). Neurodevelopmental phenotype includes epilepsy or epileptic encephalopathy, developmental delay (DD) and autism spectrum disorder (ASD).

*TRIM8* gene encodes a protein with 551 amino acids, which are involved in a broad range of biological processes, such as differentiation, proliferation, intracellular signaling, protein quality control, autophagy, and immunity (3). The protein contains a RING-finger domain, two B-boxes (B-box1 and B-box2), a coiled-coil domain, and a proline-rich C-terminal region with a nuclear localization signal (NLS) (4, 5). TRIM8 modulates the activity of important cellular proteins through protein–protein interactions mediated mainly by the coiled-coil and the C-terminus domains (6). Deletion of the C-terminal domain of *TRIM8* can result in protein mislocalization (4).

To date, a total of 18 different *TRIM8* truncating variants in 24 NRS patients have been reported in the literatures, and all of them were located in exon 6 (2, 7–14). There are no reports referring to the incidence rate of NRS. Previous studies demonstrated that there is a variability in the severity of renal or neurological phenotype. Here, we describe three previously unpublished NRS patients and provide an overview of the literatures to summarize the phenotype–genotype relationship of *TRIM8-*related NRS.

## 2 Methods

### 2.1 Patients

Three children with *TRIM8*-related NRS were recruited for this study from Peking University People’s Hospital from July 2018 to September 2023. Detailed clinical data were collected from the medical record system, including sex, family history, physical examinations, clinical manifestation (including renal and neurological symptoms), urinalysis, renal biopsy, electroencephalographic (EEG) recordings, brain magnetic resonance imaging (MRI) findings, medication, and prognosis. Follow-up was

performed by clinic visits or telephone.

### 2.2 Ethics statement

This study was approved by the Biomedical Research Ethical Committee of Peking University People’s Hospital. Written informed consent was obtained from the parents for publication of this report.

### 2.3 Molecular genetic testing

*TRIM8* gene (Ref Seq NM_030912.2) was performed using trio-based whole exome sequencing (Trio-WES) for all three patients. Sanger sequencing was performed to validate detected variations and determine their parental origin. Variants were classified as “pathogenic” or “likely pathogenic” according to the variant interpretation guidelines of American College of Medical Genetics (ACMG) guidelines (15).

### 2.4 Literature review

A systematic literature search was performed in the PubMed and HGMD electronic databases to extract relevant articles that mainly focused

on *TRIM8* variants in epilepsy or nephrotic syndrome up to December 2023. The search terms used were “TRIM8” AND (“epilepsy” or “proteinuria” or “nephrotic syndrome”). Original research, case reports, and case series were identified. Clinical

data of patients with *TRIM8*-related NRS were obtained from these identified studies.

## 3 Results

### 3.1 Case series

#### 3.1.1 Patient 1

A now-10-year-old female was born at term following an uneventful pregnancy with birth weight 3,800 g. She had no family history of renal or neurological disease. Shortly after birth, the patient was noted to have feeding difficulty. DD was subsequently observed. She could hold her head up at 5 months, sit at 11 months, crawl at 18 moths and walk unsupported at 28 months. Expressive language, especially speech, was particularly impaired, as she was only able to babble a few constant sounds. She developed afebrile hemiconvulsion at 25 months. Each seizure ceased within a few seconds to 5 min. Physical examination showed generalized hypotonia and mild facial features, including a long philtrum and odontodysplasia. She had no remarkable results of blood biochemistry, ammonia, lactate acid and metabolic disease screening. The EEGs showed marked Rolandic discharges, which was more prominent in the left region, and she was diagnosed with electrical status epilepticus during sleep (ESES). Brain MRI detected no abnormalities. Valproate acid was introduced with seizure-free for 9 months. Seizures recurred and worsened despite adding oxcarbazepine at 2 years and 10 months. Then oxcarbazepine was replaced by levetiracetam. Seizures were poorly controlled despite multiple therapies, including zonisamide, clobazam, lamotrigine and corticosteroids.

Glomerular proteinuria and mild hematuria were noted at 4 years and 5 months. Proteinuria gradually worsened (24-h proteinuria: 1.556 g) with normal serum albumin, cholesterol and creatinine. Renal biopsy revealed minor glomerular abnormalities without deposition of immune complexes by light microscopy and extensive podocyte foot process effacement by electron microscopy. After treatment with enalapril, her proteinuria decreased, but was not in complete remission (24-h proteinuria: 0.96 g).

During follow-up, seizures occurred once per year from 7 years old, all of which were induced by infective fever. Her speech delay improved due to therapy. At 10 years, she exhibited poor abilities in learning, memory and writing tasks (restricted to numbers 1–10). Her renal function remained normal up to now.

#### 3.1.2 Patient 2

A now-7-year-old female was born at term following an uneventful pregnancy with birth weight 3,150 g. During infancy, the patient was noted to have mild DD. At 1 year and 9 months, she developed focal seizures with developmental regression after seizure onset. Physical examination showed generalized hypotonia, stereotypic movements without facial deformities. Interictal EEG demonstrated slow background and diffuse spike-and-waves with ESES. Brain MRI showed periventricular leukomalacia. She had a total of three anti-seizure medications (ASMs), including levetiracetam, valproate acid and clonazepam, and continued to have occasional seizures, most of which occurred during infection.

At 1 year and 9 months, the patient was also noted to have significant proteinuria (protein 2+) and hematuria. At 6 years and 9 months, urinalysis confirmed nephrotic range proteinuria (protein 3–4+). And she developed hypoalbuminemia (26.7 g/L) and hyperuricemia. Renal ultrasound, immunoglobulins, complement, anti-nuclear antibodies and anti-double stranded DNA antibodies were all within normal limits. Renal biopsy revealed minor glomerular abnormalities by light microscopy and diffuse thinning of the glomerular basement membrane by electron microscopy. Without any treatment, her proteinuria improved (protein 2+).

By the time of 7 years old, she had no ability of walking steadily, running and jumping with fine motor regression. She could pronounce, but could not form sentences. She was incapable of understanding and executing instruction correctly. During follow-up, renal function remained normal.

#### 3.1.3 Patient 3

A now-10-year-old male was born after a normal pregnancy and delivered uneventfully. From infancy, the patient presented with delays in motor function, language, and cognition, with a significant language delay. At age of 2 years, focal motor seizure was first observed. Physical examination revealed generalized hypotonia, stereotypic movements. Interictal EEG demonstrated slow background activity and multifocal spike discharges. Ictal EEG revealed high-voltage slow waves followed attenuation, fast rhythms, resembling epileptic spasms. Urinalysis confirmed nephrotic range proteinuria with normal serum creatine and albumin. The patient also had an extensive medical history including trauma history and completed metabolic disease screening, brain MRI, chromosome karyotype, renal ultrasound and other related examinations. No other identifiable clinical or molecular abnormalities were found. Despite the application of valproate, topiramate, levetiracetam, and lamotrigine, seizures were not controlled satisfactory. Seizures were improved from 8 years old. The renal function remained normal.

### 3.2 Genetic analysis

**Figure 1:**
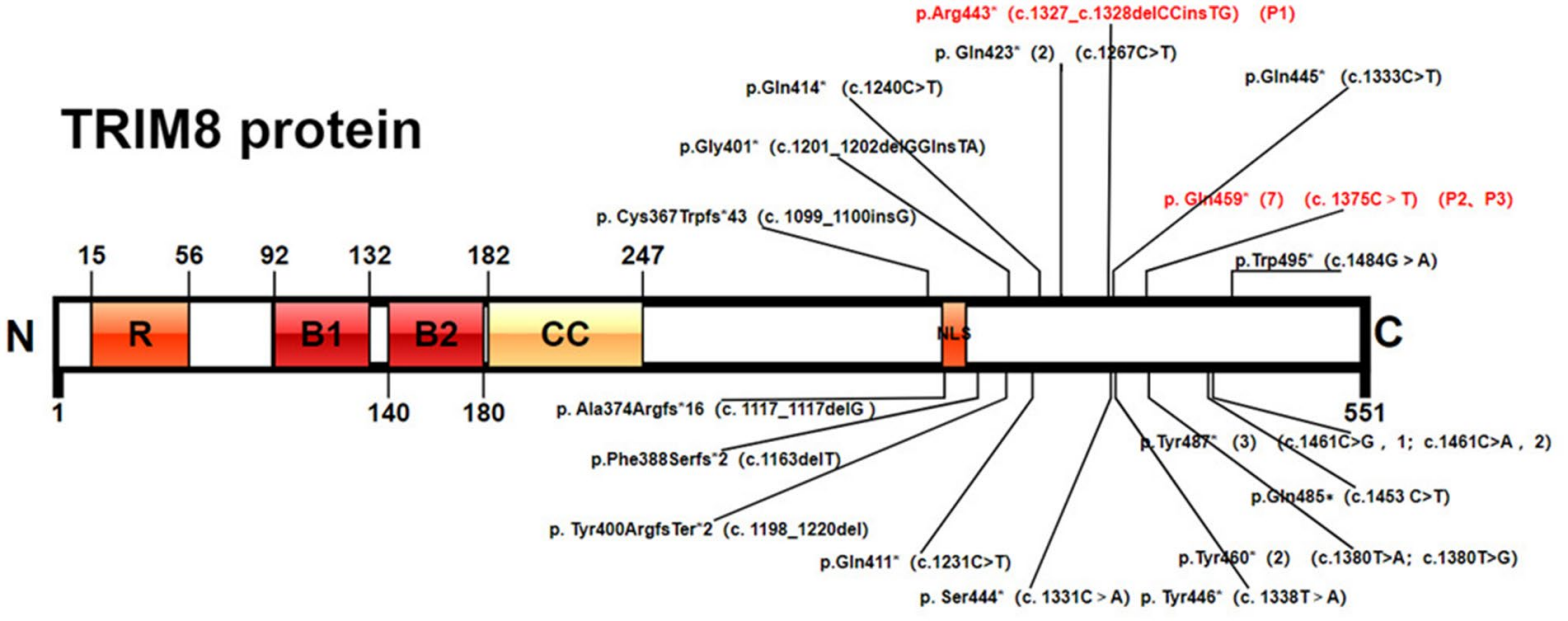
Pathogenic *TRIM8* variants. Protein domain structure of TRIM8 (NP_112174.2) and variants positions: RING-finger domain, two B-boxes (B-box1 and B-box2), a coiled-coil domain, and a proline-rich C-terminal region with a nuclear localization signal (NLS).

Two *de novo* heterozygous truncating variants were identified in *TRIM8* gene, including c.1327_c.1328delCCinsTG (p.R443*) (Patient 1), c.1375C>T (p.Gln459*) (Patients 2 and 3) (Figure 1). c.1327_c.1328delCCinsTG was not reported and the other variant was reported (2, 11, 14). The variants were all located in exon 6 and lead to the early termination of protein translation encoded by *TRIM8* gene. The two variants were absent in population databases including the Exome Aggregation Consortium database (ExAC),^1^ Genome Aggregation Database (gnomAD),^2^ 1,000 Genomes (1000G).^3^ The two variants had an ACMG score of PVS1_strong + PS2_very strong + PM2_ supporting + PP3 and were classified as pathogenic variants (15).

### 3.3 Results on literature search

As of December 2023, a total of 24 patients with *TRIM8*-related NRS have been reported (1, 2, 7–14), with 18 different truncating variants of the *TRIM8* gene detected (Figure 1). The major clinical features of the 24 cases are provided in Table 1. The 24 patients include 12 males and 12 females. Among them, three patients involved only neurological diseases, two involved only renal diseases, and the other 19 involved both neurological and renal systems.

**Table 1:** Clinical features of patients with *TRIM8* variants.

Variants	Gender	Epilepsy onset (y)	Seizure type	DRE	EEG	Brian MRI	Other features	Renal feature	ERSD Onset (y)	Facial appearance	Ref
c. 1099_1100insG p. Cys367Trpfs*43	M	0.2	Focal motor seizures	YES	Atypical hypsarrhythmia evolved into focal spike discharge	Normal	DD, ASD, microcephaly	None	None	Small, upper-slanted palpebral fissures, long-arched eyebrows, thin lips, relatively large ear lobes	(8)
c. 1117_1117delG p. Ala374Argfs*16	M	0.4	Absence with myoclonias, focal motor seizures	YES	NM	Mild CS atrophy with patchy increase in T2 signal in white matter	DD, ASD, hypospadias	None	None	Long philtrum	(11)
c.1163delT p. Phe388Serfs*2	F	4.5	Generalized tonic–clonic seizures	NM	Rolandic discharges mainly in left region	Mild CC atrophy	DD	SRNS, Bx FSGS	3	None	(2)
c. 1198_1220del p. Tyr400ArgfsTer*2	M	5	Generalized seizures	NO	Normal	An old infarct in the superior aspect of the left cerebellar hemisphere	Mild DD, strabismus	SRP, hypoalbuminemia, Bx FSGS	5	None	(10)
c.1201_1202delGGInsTA p. Gly401*	F	3	Atypical febrile seizures	NO	NM	Mild CC atrophy	DD, hypothyroidism	SRNS, Bx DMS	5	None	(2)
c.1231C > T p. Gln411*	M	2.5	Generalized seizures	NM	Central left accentuated seizure pattern	Cerebral atrophy	DD, hyperopia, astigmatism, sensorineural hearing loss	SRNS, Bx FSGS	4.8	Broad forehead, plagiocephaly, high arched palate, low set ears	(2)
c.1240C > T p. Gln414*	F	2	NM	NO	NM	Cerebral atrophy	DD, hypotonia	SRNS, Bx DMS	1.1	NM	(2)
c.1267C > T p. Gln423*	M	2	Focal seizures	YES	ESES	Nonspecific T2 signal abnormalities	DD, feeding difficulty	NS, hypernatremia, Bx FSGS	12	NM	(2)
c. 1267C>T p. Gln423*	M	1.8	Generalized tonic–clonic seizures	NM	NM	Normal	DD, hypotonia	None	None	Straight eyebrows, synophrys, sunken eyes, flat, long philtrum	(11)
c. 1331C>A p. Ser444*	F	3.4	Focal motor seizures	NO	Rolandic and bilateral frontal discharges	Bilateral patchy confluent areas of T2 hyperintensity containing tiny cysts in the peritrigonal and frontal white matter	DD, ataxia	NRP	None	None	(11)
c.1333C > T p. Gln445*	F	1.5	Tonic–clonic seizures	NM	NM	Posterior fossa dilatation and cerebral atrophy	DD, spastic dystonic quadriplegia, feeding difficulty	SRNS, Bx FSGS	19.7	None	(2)
c. 1,338 T>A p. Tyr446*	F	2	Generalized tonic–clonic seizures, tonic seizures, atypical absence, head drops, infantile spasms	YES	Multifocal repetitive spike–wave activity	Delayed myelination	DD, hypotonia, feeding difficulty, stereotypic behavior, ataxia	SRNS, Bx mesangial glomerulonephritis with IgM deposits	None	Gingival overgrowth, synophrys, long philtrum	(11)
c. 1375C>T p. Gln459*	F	None	None	NM	Rolandic discharge (ESES)	Hypomyelination at the anterior limb of the bilateral internal capsule and the bifrontal lobes white matter	DD, hypotonia, hyporeflexia, microcephaly	Proteinuria, spontaneously resolved	None	None	(11)
c. 1375C>T p. Gln459*	M	2	Focal motor seizures	NO	Slow background, Rolandic discharge (ESES)	Mild cerebral atrophy	DD, hypotonia, ASD	SRNS, Bx FSGS	5.3	Broad nasal bridge, bilateral low set ears, widespread nipples	(2)
c. 1375C>T p. Gln459*	M	2.5	atypical absences, atonic seizures	NO	NM	normal	DD, ASD, microcephaly	NRP, hypertension, Bx FSGS	9.8	None	(2)
c. 1375C>T p. Gln459*	F	2.5	NM	YES	NM	mesial temporal sclerosis	DD, astigmatism, amblyopia, Pseudostrabismu-s, hypothyroidism	NS, Bx FSGS	5	Micrognatia	(2)
c. 1375C>T p. Gln459*	M	2.5	generalized tonic–clonic seizures	NO	normal	normal	DD, ASD, hypothyroidism	SRNS	None	None	(14)
c.1380 T > A p. Tyr460*	M	2.4	focal tonic–clonic seizures	YES	NM	normal	DD	SRNS, Bx FSGS	>5	None	(12)
c.1380 T > G p. Tyr460*	F	1.5	focal seizures	NO	NM	CCB atrophy	DD, hypotonia	NRP, Bx FSGS	8	None	(2)
c.1453\u00B0C > T p. Gln485∗	F	None	None	None	NM	NM	None	SRP, mild hematuria, Bx glomerular segmental mesangial proliferative lesions with IgA deposit	None	None	(13)
c.1461C > G p. Tyr487*	M	None	None	None	NM	NM	Mild DD, hypotonia, Tourette’s syndrome-like symptoms, ASD	SRNS, Bx FSGS	14	NM	(2)
c.1461C > A p. Tyr487*	F	None	None	None	Normal	normal	None	NS, polyuria, polydipsia, Bx FSGS and cystic dilatation of distal tubules	8	None	(7)
c.1461C > A p. Tyr487*	F	None	None	None	Normal	Normal	Mild DD	SRNS, hypertension (convulsion twice), Bx FSGS	6	None	(13)
c.1484G > A p. Trp495*	M	None	None	None	NM	Bilateral frontal extracerebral space widened, left ventricle fuller than right ventricle, an arachnoid cyst in the cisterna magna	Neurogenic bladder (relieved by Solifenacin), astigmatism, myopia	SRNS, Bx FSGS and cystic dilatation of renal tubules	6	Low-set ears, micrognathia, thin lips, wide eye distance, stubby neck, and large ear lobes	(9)
Our patient											
c.1327_c.1328delCCinsTG p. Arg443*	F	2.1	focal motor seizures	YES	Rolandic discharge (ESES)	Normal	DD, hypotonia, feeding difficulty	NRP, Bx minimal change disease	None	Long philtrum, odontodysplasia	P1
c. 1375C>T p. Gln459*	F	1.8	focal motor seizures	YES	Slow background, diffuse spike-and-waves (ESES)	Periventricular leukomalacia	DD, stereotypic behavior, hypotonia	NS hyperuricemia, Bx minimal change disease	None	None	P2
c. 1375C>T p. Gln459*	M	2	Infantile spasms, focal motor seizures	YES	Slow background, multifocal spike discharge	Normal	DD, hypotonia, stereotypic behavior	NRP	None	None	P3

M, male; F, female; DRE, drug-resistant epilepsy; DD, developmental delay; ASD, autism spectrum disorder; NM, not mentioned; NA, not available; Bx, biopsy; CS, cortical and subcortical; CC, cerebral and cerebellar; SRNS, steroid-resistant nephrotic syndrome; SRP, steroid-resistant proteinuria; ESRD, end-stage renal disease; FSGS, focal segmental glomerulosclerosis; CCB, cerebral, cerebellar and brainstem; NRP, nephrotic-range proteinuria; ESES,electrical status epilepticus during sleep.

18 (75%) of 24 patients developed epilepsy with seizure onset ranging from 0.2 to 5 years. Three patients presented various seizure types during the course of the disease, including focal motor seizures, tonic seizures, generalized tonic–clonic seizures, absence with myoclonias, atypical absence seizures, atonic seizures and epileptic spasms. Interictal EEGs varied from normal, to focal discharges, Rolandic discharges, slow background with multifocal spike discharges, and atypical hypsarrhythmia. Three patients reached ESES and one patient had no seizure attack. Brain MRI changes documented in 15 (68%) of 22 patients with available data included varying degrees of brain atrophy (*n* = 8), hypomyelination (*n* = 2), mesial temporal sclerosis (*n* = 1). In addition, drug-resistant epilepsy occurred in 6 of 14 (42%) patients with available data on anti-epilepsy treatment. Furthermore, DD (*n* = 21), hypotonia (*n* = 7), autism spectrum disorder (*n* = 6), feeding difficulty (*n* = 3), microcephaly (*n* = 3), ataxia (*n* = 2), neurogenic bladder (*n* = 1) and spastic dystonic quadriplegia (*n* = 1) were observed.

21 (87.5%) of 24 patients developed renal manifestation including asymptomatic proteinuria, nephrotic-range proteinuria or typical nephrotic syndrome. 16 (16/21, 76%) patients eventually progressed to end-stage renal disease occurred at 1.1 to 19.7 years. Renal biopsies were performed in 18 patients: two had mesangial glomerulonephritis, two had diffuse mesangial sclerosis, 14 had FSGS.

Specific facial features were observed in eight patients including long philtrum, straight eyebrows, sunken eyes, micrognathia, broad forehead, broad nasal bridge, upper-slanted palpebral fissures, thin lips, large ear lobes, and low-set ears. In addition, astigmatism/myopia/amblyopia/pseudosquinting (*n* = 4) and hypothyroidism (*n* = 3) were observed.

All 18 *TRIM8* variants were truncating variants, which located in exon 6. Among 24 patients, five individuals shared the identical variant at position Gln459* (c.1375 T>C), three at position Tyr487* (c.1461C>A/G), two at position Tyr423* (c.1267C>T) and two at position Tyr460*(c.1380 T>A/G).

## 4 Discussion

*TRIM8*-related NRS is an autosomal dominant disorder caused by truncating variants of the *TRIM8* gene, with only 24 patients and 18 genetic variants reported to date (2, 7–14). In the present study, we report three patients with *de novo TRIM8* truncating variants. By combining 24 patients in previous reports, a total of 27 patients with *TRIM8*-related NRS are reviewed herein, better understanding the clinical characteristic in children.

The most common neurological presentations included DD (89%) and epilepsy (78%). In the patients with clinical information, focal seizure was the most frequent seizure type (57%). 19% of patients developed various seizure types during the course of the disease, one of whom could be diagnosed as Lennox–Gastaut syndrome. 52% of patients presented drug-resistant epilepsy among the 17 patients with available data on anti-epilepsy treatment. Our three patients all had intractable seizures with 3 to 6 anti-seizure medication. With ongoing brain maturation, improvements in seizure activity were found. DD was noted before or after seizure onset, and developmental regression was found in some patients. This phenomenon suggested that developmental impairment was due to both the etiology and superimposed epileptic activities. Language impairment was the most frequent concern in our three patients. The implementation of speech and language therapy is crucial for improving the prognosis. EEGs exhibited no specific abnormalities. It is worth noting that four patients developed ESES, three patients of whom shared the identical variant at position Gln459*. Among 25 patients whose neuroimaging information could be obtained, 64% exhibited non-specific brain MRI abnormalities. Brain atrophy was the most common change (50%).

Renal diseases are the most recurrent feature of NRS (89%). 67% of patients eventually progressed to ESRD. FSGS was observed in 70% of patients who underwent renal biopsy. The present three patients developed nephrotic-range protein or nephrotic syndrome with normal renal function. Renal biopsies were performed for two patients. Both revealed minimal change disease, which suggested that renal pathology had the potential to serve as a prognostic indicator for long-term outcomes. Renal diseases occur at the same time as or after nervous system diseases. Therefore, a routine urinalysis is necessary for epileptic patients.

Specific facial features were observed in 33% patients, including long philtrum, straight eyebrows, sunken eyes, micrognathia, broad forehead, broad nasal bridge, upper-slanted palpebral fissures, thin lips, large ear lobes, and low-set ears. In addition, other clinical features could be observed, including astigmatism/myopia/amblyopia/pseudosquinting and hypothyroidism. Evaluation of other organs, including eye and thyroid, is essential in *TRIM8*-related NRS patients.

Altogether, 19 *TRIM8* truncating variants were detected including nonsense variants (79%) and frameshift variants (21%). All variants clustered within the last exon between residues 367 and 495 of the 551 amino acid protein. Reviewing 27 patients demonstrated that seven individuals shared the identical variant at position Gln459*, three at position Tyr487*, two at position Tyr423* and two at position Tyr460*. Two patients reported by Sakai et al. (Cys367Trpfs*43) and Assoum et al. (Ala374Argfs*16), which were closer to the N-terminal, had drug resistant epilepsy, developmental delay and ASD, but without renal damage (8, 11). Five patients with variants loci at Gln485∗, Gln487∗ and Gln495∗, which were closer to the C-terminal, had no seizure attack (2, 7, 9, 13). Among the five patients, two had mild DD and one had neurogenic bladder, which suggested slight involvement of the nervous system. The recurrent variant Gln459* was the most common variant of *TRIM8*, which was found in 7 of 27 patients (25.9%). Six patients developed epilepsy and DD, while the remaining one was diagnosed with ESES without seizure attack. One patient reported by Sun X et al. had seizure spontaneously relieved without anti-seizure medication (14). Both our patients had drug-resistant epilepsy. Similarly, the severity of the renal phenotype was variable, ranging from proteinuria spontaneously resolved to ERSD during follow-up. It suggested that phenotype with the same variant locus might differ in their severity.

TRIM8 or RING finger protein 27 (RNF27) is encoded by the *TRIM8* gene which positioned on the chromosome 10q24.32. TRIM8 protein is ubiquitously expressed in various adult human tissues, especially localized to the nuclear bodies of renal podocytes and neuronal cells. Hence the main clinical features of NRS include the renal and neurodevelopmental phenotype. TRIM8 interacts with the suppressor of cytokine signaling-1(SOCS-1), which is mediated by the C-terminal region. Co-expression of TRIM8 with SOCS-1 decreases the stability and thus the levels of SOCS-1. The reduced expression of SOCS-1 correlates with decreased inhibition of IFN-*γ*-induced JAK–STAT activation (16). Both Warren et al. and Shirai et al. reported the observation of strong staining of SOCS1 protein with anti-SOCS1 antibodies in kidney from their patient, which confirmed this mechanism (7, 12). However, the relationship between the TRIM8, SOCS1 protein expression, and renal manifestations remain unknown. Furthermore, wild-type TRIM8 localized to discrete nuclear bodies, while TRIM8 bearing truncating variants in the C-terminus mis-localized diffusely to the nucleoplasm (4). However, the cellular role of endogenous TRIM8 in these nuclear bodies remains unknow.

## 5 Conclusion

This study reported one novel truncating variant, expanding the variants spectrum. TRIM8 protein is highly expressed in renal podocytes and neuronal cells. *TRIM8* gene mutation should be cautioned in children with epilepsy, DD, and proteinuria. Gln459* variant is the most common variant. Neurological and renal phenotype with the same variant locus may differ in their severity. Further research is needed to explore the relationship between genotype and phenotype of *TRIM8* variants.

## Data Availability

The data presented in the study are included in the article/supplementary material, further inquiries can be directed to the corresponding author/s.

## Footnotes

^1^ http://exac.broadinstitute.org

^2^ http://gnomad.broadinstitute.org

^3^ http://asia.ensembl.org/Homo_sapiens/Info/Index
